# Communication aspects of feedback from workers’ health surveillance due to hand-arm vibration exposure − a scoping review

**DOI:** 10.1186/s12995-025-00463-8

**Published:** 2025-05-21

**Authors:** Catarina Nordander, Mats Hagberg, Eirik Reierth, Tohr Nilsson

**Affiliations:** 1 Department of Laboratory Medicine, Division of Occupational and Environmental Medicine, Lund University, Lund, Sweden; 2 Institute of Medicine, Sahlgrenska Academy, Occupational and Environmental Medicine, University of Gothenburg, Gothenburg, Sweden; 3 Science and Health Library, University Library, UiT The Arctic University of Norway, Tromsø, Norway; 4 Department of Public Health and Clinical Medicine, Section of Sustainable Health, Occupational and Environmental Medicine, Umeå University, Umeå, Sweden

**Keywords:** Occupational health services, Workers' health surveillance, Public health surveillance, Occupational health, Health communication, Employee health, Communication, Sender, Receiver, Content

## Abstract

**Background:**

The feedback of the surveillance results to the employee and the employer largely determines the impact of workers’ health surveillance on workers’ health and exposure. We are unaware of any guidebooks or articles on performing feedback on regulated workers’ health surveillance, e.g., for vibration-exposed workers.

**Objectives:**

To identify existing knowledge of the communication aspects related to workers’ health surveillance feedback in hand-arm vibration exposure, considering the perspectives of employees, employers, and groups.

**Eligibility criteria:**

We followed the extension for the Scoping Reviews (PRISMA-ScR) checklist. No time limits were set, so the databases were searched from their start (MEDLINE 1946 and EMBASE 1947) until the date of the full search (March 2024). Relevant information was extracted from 30 articles—none concerned hand-arm vibration but covered aspects of workers’ health surveillance feedback.

**Sources of evidence:**

Two authors screened all abstracts in random pairs. They were blinded to each other’s results. The third author resolved conflicts. Inclusion criteria were full-text articles, humans, workers’ health surveillance, and aspects of communication reporting results to the employee, the workplace, or a health surveillance system. Altogether, 1914 abstracts were screened, and 84 full-text articles were assessed, of which 54 were excluded as they did not fulfill the criteria. The final publications selected included 30 articles published between 1980 and 2023; two blinded authors extracted relevant information in random pairs.

**Results:**

We found 16 of the included studies of longitudinal design, seven qualitative studies, four studies were cross-sectional, and three publications were reviews. The studies reported on workers’ health surveillance that addressed musculoskeletal disorders and pain (*n*=8), risk of cardiovascular disorders (*n*=4) or hearing disorder (*n*=3), workability and fitness for duty (*n*=3), mental health (*n*=2), allergy/ asthma (*n*=2), and cancer (*n*=1). Additionally, seven studies addressed a mixture of disorders and general health (*n*=7).

**Conclusions:**

No publications addressed communication in workers’ health surveillance due to hand-arm vibration exposure. However, we identified 30 studies addressing feedback from workers’ health surveillance that were also relevant to workers’ health surveillance due to hand-arm vibration exposure.

**Supplementary Information:**

The online version contains supplementary material available at 10.1186/s12995-025-00463-8.

## Background

To ensure member states to increase workers’ health surveillance, the European Union (EU) published the Council Directive 89/391 on 12 June 1989 on worker’s health surveillance required for harmful exposure to different types of agents in the European Union [1]. Physical agents such as noise and vibration are examples of such exposures. Chemical, bacterial, manual material handling, and night shift work are other agents. The employer must perform an employee risk assessment to determine what workers’ health surveillance is needed. The work environment authorities must regulate and inspect that the proper workers’ health surveillance is performed.

The impact of workers’ health surveillance on workers’ health and exposure is largely determined by the feedback of the surveillance results to the employee and the employer. Furthermore, feedback to government (e.g., work environment authorities) may be important for determining the need for and types of inspections of work sites and threshold limits of exposure.

An EU-OSHA report “Alert and sentinel approaches for the identification of work-related diseases in the EU European Risk Observatory Report” 2018 [2] stated that

“Individual sentinel signals are mainly used to raise alerts and trigger preventive actions at the workplace level. However, if the signal is strengthened, they can also be used to alert occupational and public health authorities.”

Currently, the European Union has not specified how feedback from workers’ health surveillance should be done. We are unaware of guidebooks or papers on how this feedback should be performed. However, it seems reasonable to assume that different aspects of communication should be considered if the feedback is directed to the employee or to the employer.

In the directive 2002/44/EC – vibration [3] it is stated that:

“Member States shall ensure that in case of a positive diagnose the worker is informed immediately and receives any required information and advice and that the employer reviews the risk assessment. Member States must establish arrangements to ensure that health records are made on individual basis that can be consulted by the workers.”

Our original aim with the scoping review was to assess the knowledge of feedback of health surveillance of hand-arm vibration syndrome to the employee and employer. However, since we did not find such an experience published, we widened the scope to consider general feedback on workers’ health surveillance. Thus, the objective was to assess existing knowledge of the communication aspects related to workers’ health surveillance feedback by a scoping review.

## Methods

Our reported items follow the extension for Scoping reviews (PRISMA-ScR) checklist [4] and used the JBI´s concepts (population, concept, context) in reporting eligibility criteria [5]. The electronic database search was preceded by concept analysis and test searching. The search used a building block strategy with Boolean operators and, in addition, a manual search that covered the lists of references in retrieved eligible articles. No search filter or limit was used to avoid losing publications that were not indexed. Our research question guided us toward selecting the databases Medline and Embase.

### Concepts

#### Feedback

This scoping review aims to identify the existing knowledge of the communication aspects related to feedback from workers’ health surveillance. Feedback in communication aims to comprehend the result of the information given. Feedback is a concept used in many disciplines. In rehabilitation feedback on movement from sensors, i.e., EMG, or therapists, i.e., manual or auditory feedback, may improve sensorimotor disturbances [6]. In nutrition health surveillance computer tailored personalized written or auditory feedback can change behaviour of fruit and vegetable consumption [7]. In occupational health surveillance, the target of feedback is most often a change of behaviour that leads to less potentially harmful exposure, e.g., work-related stress [8]. The feedback has, in various settings, been provided as individual feedback, group feedback, or as a combination of both. The construct “A feedback method” and its application have successfully been defined, practiced, and evaluated concerning prevention of back pain [9]. The feedback concept used throughout this review is restricted to reporting results from medical health surveillance and the way in which feedback is presented to the employee and employer. Articles should address an information transfer process to be eligible for selection.

#### Workers’ health surveillance

The concept of “workers’ health surveillance” is central in ILO (International Labor Organisation Geneva Switzerland), in NIOSH (National Institute of Health Morgantown, USA), in EU-OSHA (European Agency for Safety and Health at Work Bilbao Spain) and in HSE (Health and Safety Executive UK, London). The common ground is that monitoring workers health and the exposure at work can detect health effects related to work. The employer can act to reduce or eliminate risks detected. In most countries there are legal requirements for workers health surveillance. In the EU (European Union) member states are obliged to have medical surveillance of workers exposed to hand-arm vibration. The directive 2002/44/EC [3] states:

“In any event, workers exposed to mechanical vibration in excess of the values stated in Article 3(1)(b) and (2)(b) shall be entitled to appropriate health surveillance.”

#### Communication

The concepts defining communication come from a variety of interdisciplinary domains [10] ranging from e.g. technology to philosophy [11]. The present models of human communication use predominantly sociological and psychology-orientated concepts referring to communication theories with a cognitive emphasis [11] including encoding, attention, perception, understanding, memory and decoding.

The definition of communication, as used in this review includes the process of passing information and understanding from one person to another [12] or to a group of persons. The communication process may be categorized into a series of components constituting a chain of communication; Sender or source, Information content or message, Mode of communication or channel, Receiver, listener, or recipient, Context or environment [13]. In addition, the communication process may be interfered by communication barriers such as physical barriers (e.g., geographic distance or noise), language barriers (language, technical terms, lack of clarity), cultural barriers (norms, beliefs, nonverbal cues), emotional and psychological barriers (emotional state, anxiety, stress, biases, lack of motivation or lack of attention/memory or time lapses challenging memory) interpersonal barriers (communication styles), or organizational barriers.

When extracting the significant communication aspects presented in the finally selected articles we used the following concepts to categorise the communication of results from the health surveillance examinations: “The content” (results regarding and Individual or a Group) transfer from “The sender” to “The receiver” in text or in a dialog, orally face-to face, summarized as “The mode” of communication with distinction between “The receiver” being a sole employee, an employer, or a number of employees as a group. In addition, we extracted information on “The timing”, “The location”, and “Other”.

#### Context and population

Selected articles had to report a feedback process from health surveillance, restricted to a job or an occupational setting. Work, job, or occupation was necessary criterion for the article to be selected. The populations covered were individual employees, employers, and groups of employees.

### Literature search

A literature search strategy was developed, and the full search was performed on the March 24th, 2024, in the following databases: Ovid MEDLINE(R) and Epub Ahead of Print, In-Process, In-Data-Review & Other Non-Indexed Citations, Daily and Versions, and Ovid Embase Classic + Embase. No time limits were set, so the databases were searched from their start (MEDLINE 1946 and EMBASE 1947) until the date of the full search.

In collaboration, the senior academic librarian (ER) developed the search strategy from a list of key search terms and keywords to a more advanced concept map based on aim and inclusion criteria. Search blocks were created and conditionally combined using Boolean operators because of the results from pilot testing. The process allowed for iterative refinement of the concepts. The final search strategy consisted of four blocks (presented in supporting information Supplementary Fig. 1). Each block included, e.g., controlled search terms (MESH/EMTREE index terms), synonyms, and keywords. A screenshot of the search string is presented in Supplementary Fig. 2. The primary block addressed “hand-arm vibration and health surveillance,” supplemented with a block on “aspects of communication” and one on “management.” All are conditioned to a block on the domain of work or job setting (“context”). The search accuracy was tested on a compiled list of exemplar articles. Finally, ER translated and customized the concepts for indexing in the two databases used. Sampling precision was checked against manual search.

The search strategy followed the Preferred Reporting Items for Scoping reviews (PRISMA Scr). We used the controlled vocabulary of Medical Subject Headings (MeSH) from MEDLINE, and the Emtree thesaurus from EMBASE when applicable. In addition, we performed free text searches in the search fields, title, abstract, and keywords, and scanned reference lists of articles that were included for references of interest (citation search/hand search). Further, manual search on google search was performed but did not add uncaptured articles.

We exported all references to Endnote TM (97.4, Thompson Reuters. Toronto, ON, Canada), removing duplicates. All references were then imported to Covidence (a web-based collaboration software platform that streamlines the production of systematic and other literature reviews https://www.covidence.org/) and screened.

Two authors screened all abstracts in random pairs (MH, TN, and CN). They were blinded to each other’s results. The third author resolved conflicts. Inclusion criteria were full-text articles, humans, workers’ health surveillance, and some aspect of communication reporting results to the employee, the workplace, or a health surveillance system. Articles not written in English, Swedish, Norwegian, or Danish were excluded. At this stage, articles with abstracts that did not fulfill the criteria were excluded. For the rest, full-text articles were retrieved and assessed for eligibility. The same inclusion and exclusion criteria were used, and again, the articles were assessed by two blinded authors in random pairs, and the third author resolved conflicts.

## Results

**Fig. 1:**
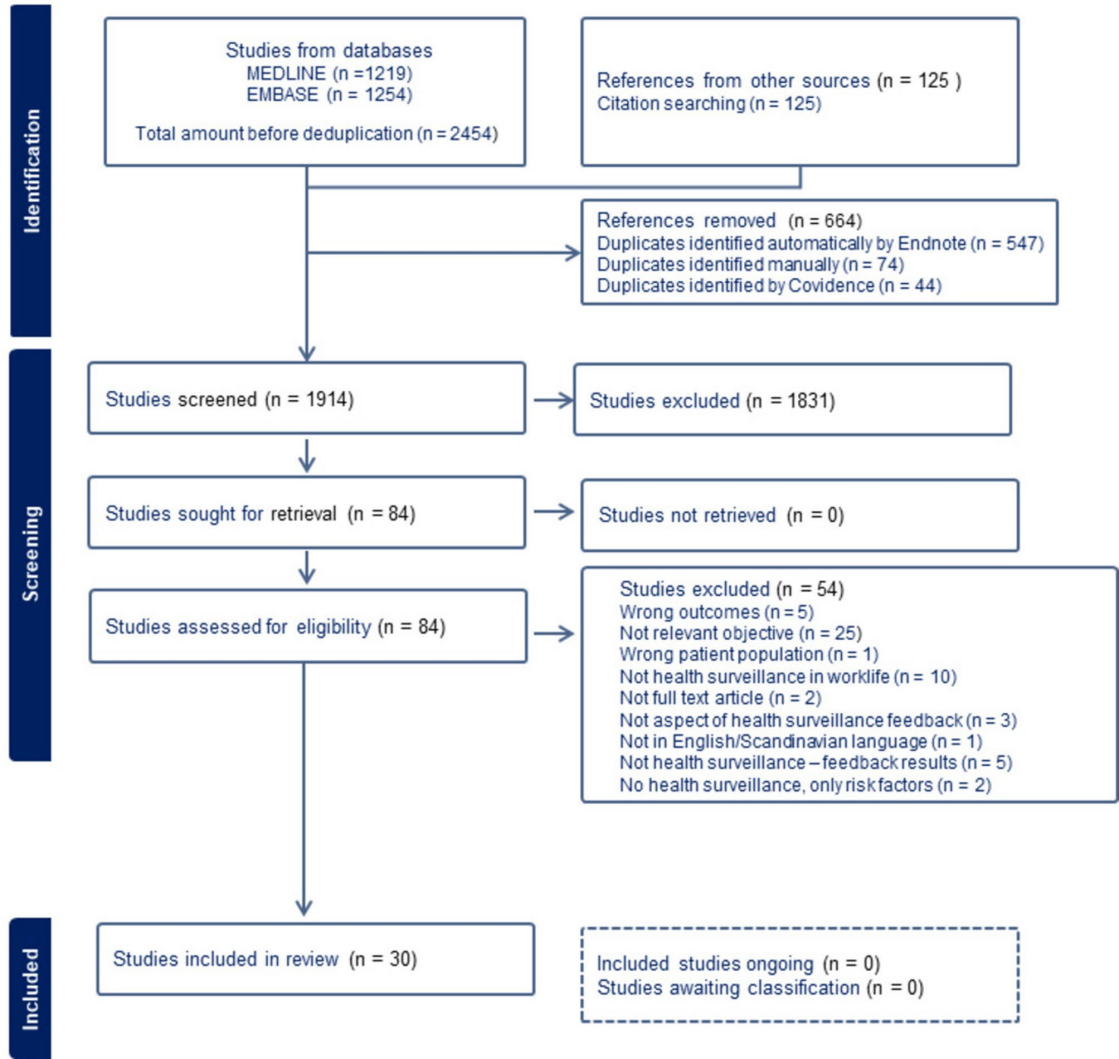
Flow chart for search strategy and selection of studies

The reference lists in the selected articles were screened, and 125 references that could be of interest were imported to Covidence, and the procedure was followed. Altogether 1914 abstracts were screened (Fig. 1).

Eighty-four full-text articles were assessed, of which 54 were excluded as they did not fulfil the criteria (Fig. 1). From the remaining 30 articles, an extraction of relevant information was made by two blinded authors in random pairs. Apart from first author, title, and year of publication the following information was extracted: type of study (qualitative, cross-sectional, longitudinal or review); country where the study was performed; disorder (type of injury/disease); type of work or exposure and receiver of communication (employer, employee or group of employees). In addition, we also interpreted, assessed and categorised the articles based on aspects of communication [results concerning an individual or aggregated group results, the sender, the timing, the location, the content, the mode (i.e., written, oral, face to face, telephone or other), the receiver, or other aspects]. More than one aspect could be indicated. Further, we extracted relevant results, conclusions, and key points, as well as other relevant comments from the studies. Finally, all three authors compared these extractions and decided on which information to report.

### Included studies

**Table 1:** Descriptive characteristics of the 30 selected studies. Aspects of communication including whether the reported results concern an individual or aggregated results from a group, when applicable

Author Year Receiver of communication	Title	Type	Country	Disorders	Type of work or exposure	Aspects of communication
Boschman 2013a [14] Employee	Preventive actions taken by workers after workers’ health surveillance: a controlled trial	Longitudinal	The Netherlands	Work-related health problems, reduced work capacity and/or reduced work functioning.	Bricklaying and supervision	Sender; Content; Mode; Receiver Individual
Boschman 2013b [15] Employee	Improving occupational health care for construction workers: a process evaluation	Longitudinal	The Netherlands	Various	Construction workers	Sender; Location; Content; Mode Individual
Codling 2017 [16] Employee Employer	Current practices in noise health surveillance An exploratory study on the delivery of noise health surveillance programmes in Britain	Qualitative	UK	Hearing loss	Noise	Sender; Timing; Content; Mode; Other Individual; Group
Di Battista 2019 [17] Employee	Cardiovascular risk assessments at occupational health services: employee experiences	Qualitative	UK	Cardiovascular risk factors	Hospital and steel work	Sender; Timing; Mode
Eklöf 2004 [18] Employee Employer Group	Feedback of workplace data to individual workers, workgroups or supervisors as a way to stimulate working environment activity: a cluster randomized controlled study	Longitudinal	Sweden	Self-reported physical complaints ≥ 3 days during the past month (neck, shoulders, arms, hands or lower back; eye complaints; headache)	Ergonomic: working hours at computer; number of not-optimal workplace design aspects; number of not-optimal working technique aspects, Psychosocial: job demands, job control, social support	Sender; Timing; Content; Mode Individual; Group
Eklöf 2006 [19] Employee Employer Group	Are simple feedback interventions involving workplace data associated with better working environment and health? A cluster randomized controlled study among Swedish VDU workers	Longitudinal	Sweden	Musculoskeletal symptoms or eye discomfort	Workplace design and psychosocial factors: working time/week, % of working hours at computer, number of not optimal workplace design aspects, working techniques	Sender; Timing; Content; Mode Individual; Group
Eliasson 2021 [20] Employee Employer	Company representatives’ experiences of occupational health surveillance for workers exposed to hand-intensive work: a qualitative study	Qualitative	Sweden	Pain and disability from neck and upper extremity	Hand-intensive work	Sender; Location; Content; Mode; Receiver Individual; Group
Eliasson 2022 [21] Employee Employer	Ergonomists’ experiences of executing occupational health surveillance for workers	Qualitative	Sweden	Musculoskeletal disorders	Hand-intensive work	Sender; Location; Content; Mode; Receiver Individual; Group
Fishwick 2016 [22] Employee Employer	Health surveillance for occupational asthma in the UK	Cross-sectional	UK	Asthma	Bakeries, wood working and motor vehicle repair	Sender; Content; Mode; Receiver Individual
Franco 2002 [23] Employee Employer	From risk-based health surveillance to health promotion: An evidence-based experience in a health care setting	Longitudinal	Italy	Musculoskeletal disorders and accidents; irritative and allergic local or general disorders; infectious diseases and biological accidents; gastrointestinal changes; sleep disturbances; chronic fatigue	Musculoskeletal load; biological agents; chemicals stress; shift work	Content; Receiver; Other
Groeneveld 2008 [24] Employee	Design of a RCT evaluating the (cost-) effectiveness of a lifestyle intervention for male construction workers at risk for cardiovascular disease: The Health under Construction study	Longitudinal	The Netherlands	Cardiovascular disease risk score (derived from periodic health screening results: questionnaire and physical examination)	Construction work	Individual Timing; Content; Mode; Receiver
Grooten 2016 [25] Employee	Health risk appraisals in Swedish occupational health services	Qualitative	Sweden	Self-reported questionnaire data on health: musculoskeletal symptoms, stress related symptoms	Lifestyle factors: working conditions: ergonomics, stressors, physical environment, organization	Content; Mode
Hanlon 1998 [26] Employee	Behaviour change following a workplace health check: how much change occurs and who changes?	Longitudinal	UK	General health	An engineering factory, white- and blue-collar workers	Sender; Timing; Content; Mode; Receiver Individual
Hubbell 2022 [27] Employee	Factors linked to participant attrition in a longitudinal occupational health surveillance program	Longitudinal	USA	Cancer, lung illness, kidney issues, diabetes, cardiovascular, hearing loss	Department of energy - hazard materials	Timing; Content; Mode; Receiver Individual
Ketelaar 2013 [28] Employee	Mental Vitality @ Work—A workers’ health surveillance mental module for nurses and allied health care professionals. Process evaluation of a randomized controlled trial	Longitudinal	The Netherlands	Mental health disorders	Patient work	Sender; Mode Individual
Ketelaar 2014 [29] Employee	Improving work functioning and mental health of health care employees using an e-mental health approach to workers’ health surveillance: pretest-posttest study	Longitudinal	The Netherlands	Questionnaire data on: stress, work-related fatigue, risky drinking behaviour, depression including suicide risk, anxiety including panic disorder, and posttraumatic stress disorders	All nurses, including surgical nurses and anaesthetic nurses, and allied health professionals (such as physiotherapists and radiotherapists) employed at one academic hospital	Timing; Content; Mode Individual
Magnavita 2023 [30] Employee Employer	Workplace health promotion embedded in medical surveillance: The Italian way to total worker health program	Review (narrative)	Italy	Various	Various	Sender; Other
Maizlish 1995 [31] Employee Employer	Surveillance and prevention of work-related carpal tunnel syndrome: an application of the sentinel events notification system för occupational risks	Cross-sectional	USA	Carpal tunnel syndrome	Ergonomic risk factors; vibration	Sender; Content; Mode; Receiver
Marsh 1991 [32] Employee	Drake chemical workers health registry study: I. Notification and medical surveillance of a group of workers at high risk of developing bladder cancer	Longitudinal	USA	Bladder cancer	Beta-naphthylamine (BNA)	Timing; Content; Mode Individual
Menckel 1997 [9] Employee Employer Group	The prevention of back injuries in Swedish health care - a comparison between two models for action-oriented feedback	Longitudinal	Sweden	Back injuries	Patient handling	Sender; Timing; Mode; Content Group; Individual
Plat 2011 [33] Employee Employer	Feasibility and acceptability of workers’ health surveillance for fire fighters	Qualitative	The Netherlands	Fitness for duty	Fire fighting	Sender; Timing; Location; Content; Mode Individual
Robroek 2012 [34] Employee	Moral issues in workplace health promotion	Cross-sectional	The Netherlands	No specific disease “Health”	Lifestyle factors physical activity, smoking, and alcohol intake BMI	Content; Mode Individual
Ruitenburg 2015 [35] Employee	Feasibility and acceptability of a worker´s health surveillance program for hospitals physicians	Qualitative	The Netherlands	Musculoskeletal disorders, stress, and depression	Hospital work, physicians	Sender; Content; Mode Individual
Seward 2001 [36] Employee Employer Group	Medical surveillance of allergy in laboratory animal handlers	Review (narrative)	USA	Allergy	Laboratory animal handlers	Content; Mode; Other Group
Soler 2010 [37] Employee	A systematic review of selected interventions for worksite health promotion. The assessment of health risks with feedback.	Review (systematic)	USA	Blood pressure, Cholesterol	A variety of worksites including manufacturing plants, healthcare facilities, health insurance companies, government offices, field settings, banks, schools, and in an ambulance service workforce.	Content; Mode: Receiver Individual
Steel 2021 [38] Employee	Disclosure in online vs. face-to-face occupational health Screenings: A cross-sectional study in Belgian hospital employees	Cross-sectional	Belgium	Musculoskeletal, skin, general mental health, respiratory, burnout and stress	Medical personnel: paramedics, technicians Administrative personnel: management, cleaning staff, others	Sender; Content; Mode
van Holland 2018 [39] Employee Employer	Effectiveness and cost-benefit evaluation of a comprehensive workers’ health surveillance program for sustainable employability of meat processing workers	Longitudinal	The Netherlands	Sickness absence, work ability and productivity	Meat packing industry	Mode; Other Individual
Verweij 2013 [40] Employee Employer	Long-term effects of an occupational health guideline on employees’ body weight-related outcomes, cardiovascular disease risk factors, and quality of life: results from a randomized controlled trial	Longitudinal	The Netherlands	Overweight	Blue-collar, white-collar, client contact	Mode
Williams 2004 [41] Employee	Does the presentation of audiometric test data have a positive effect on the perceptions of workplace noise and noise exposure avoidance?	Longitudinal	Australia	Hearing loss	Noise	Sender; Timing; Content; Mode; Receiver Individual
Zohar 1980 [42] Employee Group	Promoting increased use of ear protectors in noise through information feedback	Longitudinal	Israel	Temporary threshold shift of hearing	Noise at lathe type operations	Timing; Location; Content; Mode Individual; Group

The final publications selected included 30 articles published between 1980 and 2023 [9, 14–42]. Their descriptive characteristics are presented in Table 1. None of them studied feedback after health surveillance targeting hand-arm vibration injury, or even surveillance that was performed due to hand-arm vibration exposure. Still, we considered the results and the authors’ reflections valuable for workers’ health surveillance and therefore chose to present them.

Ten of the included studies were carried out in the Netherlands and six in Sweden, followed by the USA with five studies and the United Kingdom with four (Table 1). Two studies were conducted in Italy. Australia, Belgium, and Israel were represented with one study each.

The majority (*n* = 16) of the included studies were of longitudinal design, followed by qualitative studies (*n* = 7; Table 1). Four studies were cross-sectional, and three publications were reviews. The studies reported on workers’ health surveillance that addressed musculoskeletal disorders and pain (*n* = 8), risk of cardiovascular disorders (*n* = 4) or hearing disorder (*n* = 3), work ability and fitness for duty (*n* = 3), mental health (*n* = 2), allergy/ asthma (*n* = 2), and cancer (*n* = 1). There were additionally seven studies that addressed a mixture of disorders and general health (*n* = 7).

### Communication of results to the employee

All the 30 selected studies addressed the employee as the receiver of the feedback of the health surveillance [9, 14–42].

#### The sender

Sixteen studies focused on factors related primarily to the sender [9, 14–21, 26, 28, 31, 33, 35, 38, 41]. The suggested feedback provider to an individual worker depended on the disorder at focus in each specific health surveillance. For example, in studies addressing musculoskeletal problems, physiotherapists and ergonomists were the primary information providers. For discrete diseases other than musculoskeletal problems, the specific disorder guides medical personnel (physicians, nurses, or audiologists) who possesses the expertise and could be an appropriate provider of the feedback.

The only question discussed concerning the sender was related to the medical legitimacy of the feedback provider. Should it be practitioners, occupational health physicians, or other medical specialists who preferably give feedback [16, 17]? In line with the professional backgrounds of the first authors of the various papers, ergonomists, physiotherapists, occupational health physicians, psychologists, audiologists, and nurses were suggested as feedback providers.

Di Battista et al. [17] highlighted the positive value of conveying trust, confidence, and a high level of knowledge and competence in the sender. A lack of confidence in the sender was observed when the risk assessment at the occupational health centre and the advice about medication from the general practitioners were contradictory. The study by Battista et al. is the only study specifically addressing personal aspects of the sender, as it highlights the importance of the sender being attentive to their own mindset and emotional state to minimize bias.

Several papers stressed a general need for the feedback provider’s competence. In the study by Boschman et al. [15], the occupational physicians reported that they needed even more knowledge on some of the preventive advice to counsel the recipient better, and that indicated that they needed more detailed training. A similar conclusion on the importance of competence of the performer of health surveillance for proper feedback was drawn by Codling et al. [16].

Menckel et al. [9] reported that the physiotherapists who delivered the feedback in their study on musculoskeletal disorders enjoyed the workers’ confidence, which was important for generating changed behaviours for prevention. Steel et al. [38] could, on the other hand, show that lower trust in the physician lowers the intention to disclose problems. Ruitenburg et al. [35] reported that when the sender was in the occupational health service, and the receiver of the feedback was a hospital physician, the latter presented more doubts. This might, however, concern the effectiveness of occupational health surveillance or a fear of medicalization among the hospital physicians.

There were altogether few studies that analysed the impact of factors specifically related to the sender of feedback and how this influences an effective perception of the feedback information.

#### The timing

Thirteen studies reported aspects on the importance of timing on when to give feedback [9, 16–18, 24, 26, 27, 29, 32, 33, 37, 41, 42] however, not considering that the impact of timing and choice of location are closely interrelated.

The severity of the outcome disease is suggested to determine the timing of when feedback should be given. In a screening program for bladder cancer conducted by Marsh et al. [32] they established that in the case of a positive screening result, an immediate diagnostic evaluation was warranted, together with an immediate telephone call and a written follow-up letter, while a non-negative result could be followed up within one month.

Health surveillance results providing information that causes mild anxiety has been reported as a motivating factor for behaviour change [26]. The authors suggest that when individuals receive information about their personal risk that may cause anxiety, advice and support should also be given, at the same time. This is with the pronounced intention of reducing anxiety. Other authors suggest that mild anxiety must not be avoided as fear and perceived threat might be a favourable factor for preventive change. Information received may, in addition to anxiety, for the recipient also lead to an overestimation of the risk status, e.g., concerning cardiovascular risk [37].

When risk assessment for cardiovascular disease, included e.g., HbA1c and cholesterol levels, instant feedback was given and reported. Immediate feedback was reported to facilitate engagement for prevention [17]. Urgent abnormal results were immediately notified by telephone [27], which was also reported to be a factor linked to participation attrition.

Williams et al. [41] advocated that the feedback of the hearing test results should be presented at the same session as the hearing testing. This contrasts with the delayed timing of daily feedback chosen by Zohar et al. [42]. The latter suggest repeated feedback to ensure the workers understand the significance of the feedback relative to hearing protection. Codling et al. [16], also focusing on hearing disorders, advocated feedback to the worker at the very time of testing and especially with the possibility of comparing the results from different points in time and relating them to previous assessments.

For musculoskeletal disorders, Eklöf et al. [18] regarded oral feedback to the recipient worker within one month after collecting baseline information as fulfilling the criteria of freshness of feedback information. The results highlighted the importance of the timing and location of events.

Based on the opportunities of modern digital technologies, feedback results may easily be given immediately. For example, Ketelaar et al. [29] gave all participants in an online screening program on impaired work functioning automatically generated personalized feedback, on-screen and by e-mail. However, despite immediate feedback few employees thereafter logged into the recommended digital intervention module.

#### The location

Four studies [15, 20, 33, 42] reported information on where to communicate health surveillance results. None of the studies discussed the possible effect of the physical environment or the possible value of a professional occupational health setting.

A venue for assessments and feedback close to the work site enables the possibility of early and immediate feedback, according to Plat et al. [33], who analysed the acceptability of workers’ health surveillance and discussed whether logistics were feasible and favourable. This is in line with the experiences reported by Zohar et al. [42]. They stressed the value of performing the workers’ health surveillance near their job sites, which shortened the time for reporting the audiometric tests. Thus, the choice of test site and feedback was closely related to the feedback timing. In the study by Eliasson et al. [20] the informants reported that not having to leave the workplace was time-efficient and convenient. Their experience was also that, as the ergonomist had difficulties reaching some distant workers, it could come as a surprise for the workers that they had to undergo a clinical examination, including being unprepared of the results of for the examination results.

In the Netherlands, the worker receives a designated number of hours yearly for medical purposes. The further the travel to attend a workers’ health surveillance program, the more is consumed when the examination occurs at a distant location. Thus, a distant location may be a possible barrier for workers’ health surveillance in the Netherlands [15].

#### The content

Twenty-three studies covered aspects of content [9, 14–16, 18–20, 22–27, 29, 31, 32, 34–38, 41, 42].

Maintaining confidentiality is an absolute primary requirement when selecting the content in the feedback. This holds for both when delivered to the individual worker and when extended to the employer. The negative effect of questioned confidentiality or loyalty primarily to the employer is noticed in, e.g., the paper by Steel et al. [38].

The content of the feedback may vary in terms of detail, content, framing, or the addressing of possible risks. The reported results may be presented as general aspects, e.g., cardiovascular metabolism [14, 15], summarized in a health discussion [25, 35, 38], presented as a summarized risk profile [24] or alternatively, providing workers with information about the results of the health surveillance [23, 29].

Comprehensive individual feedback of not only the disorders but also a multitude of ergonomic and psychosocial hazards combined as unspecified feedback was reported by Eklöf et al. [18].

In some countries, workers’ health surveillance aims to identify a disorder, provide treatment, and ensure an extensive hazard risk assessment. In other countries, the content of the feedback is a distinct statement on fitness for work and restriction of exposure [22, 23]. Soler et al. [37] stated, in a review, that the level of detail in the feedback may vary in relation to the communicated risk. The feedback content could be either a simple qualitative statement, a quantitative evaluation, or an individual health risk estimate. No guidance in this respect was given in the review.

The level of detail varied depending on the reported health surveillance result. Audiometry results were in several studies presented in detail [16] and the content in the hearing loss surveillance entailed feedback of high-definition details on hearing [41, 42].

Seward et al. [36] discussed in a review the neglected role of communicating medical findings back to the individual worker and suggested that analysis of aggregated data on the population of workers should be included and used to evaluate risk patterns.

Marsh et al. [32] secured that the feedback included a prompt notification of the results by using phrasing that clearly directed the listener’s attention to the possibilities of health risks without arousing unnecessary fear. Persons with screening test results in the monitor or positive categories were notified by telephone with a follow-up letter describing the test results and encouraging them to undergo semi-annual rescreening (monitor) or a free diagnostic evaluation (positive). The personal telephone contact used for the monitor and positive results has been found to be effective in reducing fear and anxiety and in permitting an opportunity for the clinical staff to respond directly to any questions that the study members may have concerning their test results.

In the study by Hubbell et al. [27] the content of feedback from the health surveillance parameters were both high in detail on separate tests (e.g., glucose level) and, at the same time, collectively summarized as an index variable. Whether the detailed result or the summarized results had higher efficacy was not discussed. It was rather those who perceived themselves as needing health change or those who worried about the information that made a healthy lifestyle change.

Comparing the outcomes with former results and repetition of the results from the workers’ health surveillance was advocated in several reports to increase the preventive effect [16, 24, 26, 42].

Hanlon et al. [26] aimed to analyse the value of the different amounts of feedback (feedback of cholesterol, cholesterol with risk assessment, and in addition a health education) on how many workers actually comply with behaviour change after receiving feedback and advice. Almost half of the participants responded positively and those at risk complied with a large extent to the information. Grooten et al. [25] reported that, as to their knowledge, no studies have explored the amount, extent, and nature of individual feedback (evaluation and suggestions for improvement) by the occupational health service and what is perceived by the receiver of such feedback.

Results from health surveillance have traditionally concerned injury or loss of function, i.e., loss-framing. The hearing-loss study by Williams et al. [41] concluded that messages may need to be framed more positively to gain positive preventive results. A similar conclusion was drawn by Zohar et al. [42] who showed that individual feedback motivation was superior to enforcement of disciplinary actions. The importance and advantage of choosing gain-framing instead of loss-framing on attitudes and participation was reported in several studies. Zohar et al. [42] could show the positive effect of earplug use motivated by gain-framing. Williams et al. also discussed this finding [41], referring to a publication by Detweiler et al. [43].

Irrespective of content, Eliasson et al. [20] highlighted the importance of straightforward reporting of results and paying attention to the approach and language that are used in management system frameworks within companies. The problem of adherence to recommendations and compliance was raised in several articles. The results from Robroek et al. [34] highlighted the perception of the work to be healthy and thereby not in need of intervention, followed by arguments of lack of time.

No studies were retrieved analysing the consequences of reporting false positive or negative test results.

#### The mode

Twenty-five studies [9, 14–22, 24–29, 32, 34, 35, 37–42] discussed mode of communication. In our scooping review, this was closely related to the message’s content. The mode of communicating the feedback varies from face-to face consultation [14–16, 19, 20, 26, 35, 39], written as a text document or as a pamphlet [20, 24], in some cases reported as postal letters [27], telephone [9] or as a combination of these [9, 20, 35]. In addition, electronic, on-screen feedback, or e-mail [28, 41] have been used. Aspects covered in the studies include support to cognition by using simplification, clear message, simple language, and using pictorial visualization [16, 41, 42].

The instant feedback in hearing testing has been proven favourable and face-to-face feedback with interaction has repeatedly been reported as favourable.

The results from our scooping review show that the authors usually report the way, how, and mode of communication used when they performed the feedback. Little consideration has been paid on which mode was superior, when, how, and in which context, and their possible disadvantages. Each method has its own given pros and cons [38].

The information transmitted used text printed on paper, spoken words orally face-to-face, or on a distance by telephone, or web-based digital communication with visual contact. Nineteen studies [9, 14–16, 18, 20–22, 24, 27–29, 31, 32, 35, 37, 38, 40, 42] reported on which medium was used. In most studies, the examiners chose to inform the individual worker orally, face-to-face in person [9, 14, 15, 18, 25, 26, 35, 37, 39, 40] and supplemented with follow-up on the telephone if the results were serious [32].

Text-on-paper information was used by Boschman et al. [14] who believed that written reports provided a valid method of capturing the given recommendations. Printed text [14, 15, 18], leaflets [20, 26], and letters [27] were alternately used. Sometime individuals with an overall negative screening test were notified only by letter [32], while persons with screening test results in the monitor or positive categories were initially notified by telephone and later supplemented with a more detailed follow-up letter. A personal telephone dialog was found to be effective in reducing fear and permitted an opportunity to respond directly to questions.

Ketelaar et al. [29] used computerized and online feedback. When stratified, those who felt “relatively worse” or those receiving online feed-back indicating “more impairment”, were more inclined to try online tailored advices.

The advantages of feedback with pictorial visualization were used, especially in conjunction with hearing loss surveillance [16, 41, 42] when presenting audiograms. Overhead-slide presentations was used at feedback by Eklöf et al. to stress the message [18].

#### The receiver

Seven studies [20, 23, 24, 26, 27, 37, 41] discussed preparing the receiver of the forthcoming results.

It has been assumed that it is generally favourable for a single worker to be prepared and informed of the message in forthcoming feedback. Little support is gained from our literature search concerning the specific benefits of preparing the employee for the outcomes of a worker’s health surveillance. Indirect support comes from experiences that show that not being informed is negative [20]. Williams et al. [41] reported that increasing the awareness of noise and the enclosed risks by additional testing and hearing information gave no increased hearing health benefits. From a conceptual standpoint, it is often recommended to assess readiness to change [24] and to increase awareness [20].

Several studies have paid attention to the impact of the feedback rising anxiety in the listener as a drive for change. Hanlon et al. [26] showed that those who found the feedback threatening increased the drive to change. Anxiety was also noted as an unwanted adverse side effect on the feedback [37].

Franco et [23] found that the listeners’ attention to hazards varied. However, it should be stressed that differences exist between the risk perception of different stakeholders: employers focus more on the biological risk, trade unions are more concerned about musculoskeletal load, and others consider other problems (chemicals, stress, shift work) relevant.

### Communication of results to the employer

In addition to addressing the employee as the receiver of the feedback of the health surveillance, fourteen studies also addressed the employer as the receiver [9, 16, 18–23, 30, 31, 33, 36, 39, 40].

#### The sender

Five studies addressed the sender of communication [16, 20–22, 30].

Codling et al. [16] discussed the importance of the practitioners’ awareness of the employer’s risk assessment results, in that study concerning noise exposure. Although this was well known to the practitioners, they did not always have access to such results. In those cases, health surveillance of hearing loss could be done in isolation instead of as a part of systematic risk-management of noise exposure.

Eliasson et al. [21] performed a qualitative study, and they also reported that difficulties with exposure assessments impeded the ergonomists’ work. Their work was also hampered when the work-related health status in the work group was unknown, and if there was insecurity regarding debiting for their services. From the same study, Eliasson et al. [20] discussed that ergonomists can benefit from using the same approaches and language used in the companies’ management systems for communication.

At the same theme, Fishwick et al. [22] reported a large variation in decisions regarding the requirement for health surveillance, how it was developed and carried out, and how the communication between occupational health service providers and workplaces occurred. They discussed that few occupational health services providers used a risk assessment from the worksite to design their health surveillance. Thus, the health surveillance system would have less possibility to be risk-based.

In a review Magnavita et al. [30] reported on a system for workplace health promotion with embedded medical surveillance (WHPEMS) that has been developed at the Post-Graduate School of Occupational Health in Rome and applied by occupational physicians. They used an assessment, surveillance, information, and audit model for risk management. They discussed that if crucial aspects are identified, investigations or audits must be designed so that actions for improving the work environment can be suggested. Further, they argued that an occupational physician’s intervention is fundamental for identifying workers’ health needs. They concluded that continuous health promotion, as a part of regular health surveillance in the workplace, was cost-effective and sustainable and used a participatory approach. They found it useful for collecting information on the health status of workers and for work environment improvements. They also found it a solid basis for communication between occupational physicians and the national health service doctors.

#### The content

Six studies addressed the content of the communication [16, 18–21, 33].

Codling et al. [16] discussed that it was uncommon to report anonymised grouped data on hearing loss, even though such reporting was recommended in the Health and Safety Executive guidance and the data was easy to generate. Such data could have made it easier for employers to evaluate the effectiveness of their risk management in noise reduction. The concluded that practitioners should make comparisons with previous results, provide employers with fitness for-work data, and, if possible, present group data.

Eklöf et al. [18, 19] reported that improvements in both working technique and social support could be seen after discussions about data concerning the ergonomic and psychosocial working environment.

According to Eliasson et al. [20] the results from a clinical examination of musculoskeletal disorders increased the company representatives’ knowledge of the adverse effects of hand-intensive work. As they became aware of early signs of musculoskeletal disorders, they could act immediately on such signs. Awareness of the employee rather than of the work task increases the understanding of the risks. The informants valued a detailed report focusing on the results including the current exposure levels and the prevalence of work-related musculoskeletal disorders among the workers. Further, they wanted proposals for actions that could reduce the exposure and thereby prevent musculoskeletal disorders [21].

Plat et al. [33] advised the whole fire-fighting sector to use the same materials and verbal instructions and to mandate fire fighters with positive experiences of workers’ health surveillance to help implement such surveillance nationwide.

#### The mode

One study, reported on the mode of communication. According to Eliasson et al. [20] both written and oral feedback needed to be adapted to the audience, i.e., both the employer and the workers.

#### The receiver

Three studies addressed the receiver of communication [20, 21, 31].

In the study by Eliasson et al. [20] the clinical examination surprised the workers since their employer had not informed them about the content in the workers’ health surveillance. The importance of continuous information meetings during the process with the involved project members and workers was highlighted. They discussed that a joint start-up meeting where the process was planned facilitated the hand-intense-work-model. Then, the ownership of the process, the schedule for execution and supportive management were clarified [21].

In a study by Maizlish et al. [31], it was found that though case interviews indicated symptoms and signs of exposure to uncontrolled occupational risk factors, the employers lacked knowledge, training, motivation, and expertise to reduce ergonomic risks.

#### Other aspects

Four studies discussed other aspects of communication with the employer [16, 23, 36, 39].

According to Codling et al. [16] good communication channels between the practitioner and the employer, as well as a defined scope of work (contract), were important. This should include whether the practitioner could get access to risk assessments and medical records.

According to Franco et al. [23], the health surveillance aims to ensure workers’ health by providing a medical baseline and detecting health changes at their onset. It constitutes a part of the preventive program, and it helps comply with regulations and provide fitness for job judgement. In the study, the employers stated that improved workers’ satisfaction and early detection of health changes were beneficial. They also pointed out reduced costs and improved interpersonal relations.

Seward et al. [36] reported in a review on medical surveillance of allergy in laboratory animal handlers. They found that periodic workers’ health surveillance can track the development of allergies in individuals as well as groups of workers. Thus, counselling can be improved, and disease progression can be avoided. By analysing the results on population-level analysis, systematic risk management can also be improved and means to prevent laboratory animal allergy can be evaluated.

In a study by van Holland [39] it was recommended that when studying health surveillance programs researchers should include interventions focusing both the work environment and the individual worker.

As concerns the communication with the employer, none of the studies discussed the timing or the location.

### Communication of aggregated results to a group

Five studies focused on feedback to a group [9, 18, 19, 36, 42].

#### The mode

In a cluster randomized trial by Eklöf et al. [18] they studied ergonomists’ feedback of workplace data (including their musculoskeletal symptoms and eye discomfort) to a group of workers with a supervisor present as one of four conditions. The feedback was oral face-to-face. The other conditions were individual feedback, feedback to supervisor, individual worker feedback and no feedback (control). The outcome were workplace and work technique modification. All feedback conditions differed positively from the control condition. Psychosocial modification was noted only in the feedback to supervisors.

In this feedback study, the receiver’s preparation was vital since they were informed that it was their data, and for the supervisor it was their workgroup data. The content included health surveillance data on musculoskeletal and eye symptoms. The sender was an external “consult” i.e., an ergonomist in a face-to-face meeting.

In 2006 Eklöf and Hagberg published [19] an extended analysis of the cluster randomized trial by Eklöf et al. [18]. The feedback of, among others, their musculoskeletal symptoms, eye discomfort, and psychosocial aspects was given as to group of workers with a supervisor present as one of four conditions. The other conditions were feedback to employees and supervisors and no feedback (control). Outcome variables were workplace and/or work technique modification. There was no effect on musculoskeletal symptoms and eye discomfort. The positive impact of feedback to supervisors was seen for social support. A similar effect was suggested for input to the work group. The authors discussed that the effects may be because.

“supervisors in Swedish organizations are responsible for working environment, are likely to be more aware of goals, priorities and resources, and are in a position to take decisions”.

The content included health surveillance data musculoskeletal and eye symptoms. The sender was an external “consult” i.e., an ergonomist in a face-to-face meeting. These two studies stress the importance of feedback to supervisor face to face. Feedback to group including supervisor may not be as effective as feedback to supervisor alone.

Two models of feed-back were tested to prevent back injuries in care by Menckel et al. in a longitudinal study [9]. The health surveillance consisted of notification of back injury to the occupational health service. Two models of feedback used oral face-to-face feedback, written report and video reconstruction of events leading to injury. Occupational physiotherapists gave feedback solely to the supervisor, or to the work group including the supervisor. Feedback to both supervisors and entire work groups, generated many proposals for action. In the discussion it was said that during follow-up, it proved that the proposals of supervisors had been embarked upon or completed to a greater extent.

"Of all their proposals, around 20% had been implemented within six months, and a further 20% set in motion."

In a longitudinal study, Zohar et al. [42] studied the effect of information feedback of temporary hearing losses with and without earplugs being worn in a noisy work environment. After the baseline observations of earplug use, lectures were given to groups of workers from the experimental and control departments on hearing conservation in noise.

Workers in the experimental department received additional information regarding the study’s nature. These workers were told about the audiometric testing to be conducted before and at the end of the work shift, the interpretation of such audiograms in revealing temporary threshold shifts (TTS) in hearing sensitivity, and the manner of using these TTS data as feedback to indicate the benefits of wearing the issued earplugs.

One copy of the audiogram form was given to the worker (individual feedback) and a second copy was hung on a special bulletin board in the production hall of their department for group feedback. Noted on each audiogram were the worker’s name, age, number of years of service in that department, the frequency of earplug-use up to the time of the study, and whether or not earplugs were worn during the day of the testing. At the outset of the audiometer testing feedback stage, study team members helped the workers who gathered around the bulletin board at the start of the workday or during “break” periods to interpret their fellow workers’ audiograms and apparent threshold shifts. This instruction was discontinued after all workers had become familiar with these observations and could make their own appraisal. The group feedback was displayed on the bulletin board.

The results show that the initial lectures to the experimental and control groups increased the use of earplugs. However, this was only for a few weeks in the control group. Besides the individual feedback of a temporary threshold shift, the authors also point to the group feedback on the bulletin board, which demonstrated an acceptance of earplugs by a sufficiently large number of workers in the experimental group. This created new norms and behaviour standards favouring earplug use.

The group feedback was in this study face-to-face, and individual results were publicly displayed on the bulletin board. The conclusions were:

“It is clearly seen that the feedback procedure employed in this study had a positive and sustained effect in changing worker behaviour with regard to the increased use of ear protectors … attributable to the feedback treatment.”

and

“That is, management’s attempt to use a gradual enforcement-disciplinary action plan to induce greater use of earplugs provided a limited opportunity to compare its effectiveness with that of individual feedback motivation. The latter, as the data demonstrate, is clearly superior.”

According to Seward et al. a narrative review, when training animal handlers, medical personnel should assist with training programs, and any group results or “lessons learned” from the medical surveillance effort should be incorporated into these training sessions [36].

## Discussion

In this scoping review, despite the prevalence of workers’ health surveillance, we found no publication addressing feedback from such surveillance due to hand-arm vibration exposure. However, we identified 30 studies that touched on the broader topic of workers’ health surveillance feedback. The scarcity of studies on these common activities in most countries was surprising. Most of the studies we found focused on the transference of individual test results to a single employee, with aggregated data from several single employees less frequently presented to individual employees, groups of employees, or employers.

Given the prevalence of workers’ health surveillance with feedback, there appears to be a significant opportunity for future research in this area. Most of the 30 studies we identified were longitudinal, only two were cluster randomized studies (from the same study population). This suggests that designing and successfully performing randomized (cluster) controlled studies in this context could be a feasible and fruitful avenue for further research. Such studies could potentially reveal efficient feedback strategies that could help reduce harmful exposures.

Feedback of the results was not the focus in all studies e.g. Boschman et al. [15], but lessons learned concerning aspects on communication were reported. Therefore, we extracted relevant information from the results and conclusions and from, e.g., the discussions. In this scoping review important key points were found:

### Key points for communication of results to the employee

The person who communicates results from medical health surveillance to the employee needs to have solid professional competence, possess high credibility, display confidence, and possess solid knowledge on what preventive counselling to give e.g [9, 15–17]. Immediate feedback of results increases the chance that preventive counselling is followed [16, 17, 27, 32, 41]. Communicating what can be gained by acting is more successful than reporting what has been lost [41, 42]. The employee must be assured that confidentiality is maintained unless the employee permits information on results to be communicated to others, e.g., to the employer [9, 17, 38]. Personal contact is effective in case of pathological results to reduce fear and anxiety and allow the clinical staff to respond directly to any questions the study members may have concerning their test results [29].

### Key points for communication of results to the employer

Concerning communication of results to the employer, good communication channels between the practitioner and the employer and a defined scope of work (contract) were important [16, 20, 22]This should include whether the practitioner could get access to risk assessments and medical records [16, 21, 22, 30]. A joint start-up meeting where the process was planned facilitated the risk-management model [20, 21]. Then, the ownership of the process, the schedule for execution, and supportive management were clarified. A detailed report from the health surveillance, including the prevalence of work-related disorders among the workers and the current exposure levels, was desirable [16, 18–20]. Finally, proposals for reducing the exposure were asked for [21, 30, 39].

### Key points for communication of aggregated results to a group

Health surveillance data were usually given to groups face-to-face [9, 18, 42]. These studies showed effects on work performance, use of protective equipment, and improvement proposals. Compared to group feedback, individual feedback to supervisors had additional advantages in impact and efficiency [19]. Group results or “lessons learned” from the medical surveillance effort should be incorporated into group training sessions [36]. The effect on use of personnel protective devices was less with disciplinary actions than with individual feedback motivation [42].

In the retrieved studies, we found no discussion about the possible consequences of false positive or false negative test results for employees in health surveillance feedback. For example, for vibration exposed workers if a test of cutaneous sensation shows a false positive outcome, it could result in unemployment, and if the test shows a false negative outcome, it could result in a continuation of hazardous exposure.

The original aim of this scoping review was to assess the knowledge of feedback of health surveillance of hand-arm vibration syndrome to the employee and employer. No such studies fulfilling our inclusion criteria was retrieved in the literature search. Manual search without constricts on feedback identified a few additional articles related to health surveillance of vibration exposed workers. Most of those studies focused on the imperfection in conducting health surveillance on vibration exposed workers and with less or no attention on feedback [44–50]. Some lessons from these studies deserve to be mentioned.

Poole et al. reported the results from a telephone interview with employers in the UK and found that firms are more likely to receive feedback of the results from individual workers (80%) rather than of the results on a grouped basis. Of those receiving individual feedback, 63% were forwarded to the risk management process. 10% of the employers had not received any feedback from the conducted health surveillance [49]. The authors concluded that this lack of feedback is concerning as health surveillance is unlikely to be of any value if the firm is not given information upon which it can act.

Another study in the UK on health surveillance of vibration exposure reported that every second of the occupational health providers provided incomplete health surveillance, leading to inadequate feedback to the employer [47]. These findings, combined with the lack of studies explicitly exploring the effect of feedback following health surveillance of vibration-exposed workers, underline the need for research in this field. Significant resources are invested in such surveillance, without solid knowledge on the effects.

In a position paper by Pope et al. [50] addressing health surveillance of whole-body vibration, the feedback information to the worker is suggested to include a more systematic collection of data on exposure and providing information to the exposed workers on the potential harm and the possibilities for prevention.

Only one report, an exploratory pilot intervention study retrieved after the search, addressed feedback, health surveillance and intervention concerning vibration [45]. The authors discussed aspects of intervention regarding communication from a psychological perspective, highlighting the attribution of control. They concluded that the interventionist may need extremely good knowledge of the work and its conditions. Successfully communicated intervention must address cognition and attitudes. The study identifies the importance of directing feedback and intervention to motivated workers and supervisors with sufficient self-efficacy. This emphasises our findings concerning the importance of the receiver [51].

In some countries, e.g., Italy, workers’ health surveillance of exposure to hand-arm vibration is also an examination of fitness to work [52]. This can be regarded as adequate since loss of sensation in the hands due to vibration exposure may increase the risk of accidents when handling machinery. Thus, it is not only a health issue but also a safety issue, which was stressed in several of the included studies.

## Limitations

A limitation was that few of the included studies focused on feedback communication. We, therefore, extracted not only from the conclusions but also from the discussions. We did not include all scientific databases. We believe that the included databases in combination with citation search, have captured most of the relevant literature. As the retrieved articles were spread out between many different research questions and outcomes, the reported key points are based on one or two studies.

## Conclusions

Surprisingly, no publications addressed communication in workers’ health surveillance due to hand-arm vibration exposure. However, we identified 30 studies addressing feedback from workers’ health surveillance that were also relevant to workers’ health surveillance due to hand-arm vibration exposure.

Raising anxiety in the receiver was a drive for change; those who found the feedback threatening to their health increased the drive to change. Personal contact when reporting pathological test results was effective in reducing fear and anxiety and permitting an opportunity for the clinical staff to respond directly to any questions. When comparing feedback to groups of workers with individual feedback to supervisors, the latter has additional advantages in effect and efficiency. Group training sessions should incorporate aggregated group results or “lessons learned” from the medical surveillance effort. In the retrieved studies, we found no discussion about the possible consequences of false positive or of false negative test results. It would be feasible to design randomized (cluster) controlled studies to increase the knowledge of efficient feedback procedures that reduce harmful hand-arm vibration exposure.

## Supplementary Information

Supplementary Material 1: Table. All identified studies. 54 were excluded, and 30 were included. When relevant, the reason for exclusion.

Supplementary Material 2: Fig. 1. The final search strategy consisted of four blocks at database search in Ovid MEDLINE^®^, Embase^®^ Classic and Embase^®^, conditionally combined using Boolean operators.

Supplementary Material 3: Fig. 2. Example search string Medline.

## Data Availability

No datasets were generated or analysed during the current study.
