# Supplementary material for: Communication aspects of feedback from workers’ health surveillance due to hand-arm vibration exposure − a scoping review
Source: J Occup Med Toxicol. 2025 May 21;20:16. doi: 10.1186/s12995-025-00463-8 (PMC12093587; doi:10.1186/s12995-025-00463-8)
Supplement: Supplementary file 1 — Supplementary Material 1: Table. All identified studies. 54 were excluded, and 30 were included. When relevant, the reason for exclusion. [file 12995_2025_463_MOESM1_ESM.docx]

S1 Table. All identified studies. Fifty-four excluded and 30 included. When relevant, reason for exclusion.

| # | Reference | Included/  excluded | Reason for exclusion |
| --- | --- | --- | --- |
| 1 | Addley K, Boyd S, Kerr R, McQuillan P, Houdmont J, McCrory M. The impact of two workplace-based health risk appraisal interventions on employee lifestyle parameters, mental health and work ability: results of a randomized controlled trial. Health Education Research. 2014;29(2):247-58. | Excluded | Not relevant objective |
| 2 | Aguilar KN, Smith ML, Payne SC, Zhao H, Benden M. Digital human ergonomics training for remote office workers: Comparing a novel method to a traditional online format. Applied Ergonomics. 2024;117:104239. | Excluded | Not relevant objective |
| 3 | Anderson RC, Anderson KE. Worksite health promotion. The benefits of providing personal health status feedback and education programs to employees. AAOHN Journal. 1991;39(2):57-61. PubMed PMID: 1993085. | Excluded | Wrong outcomes |
| 4 | Balmes JR. Surveillance for occupational asthma. Occup Med. 1991;6(1):101-10. PubMed PMID: 2008631. | Excluded | Not health surveillance, only risk factors |
| 5 | Biswas A, Begum M, Van Eerd D, Johnston H, Smith PM, Gignac MAM. Integrating Safety and Health Promotion in Workplaces: A Scoping Review of Facilitators, Barriers, and Recommendations. Health Promotion Practice. 2022;23(6):984-98. | Excluded | Not health surveillance in work life |
| 6 | Boschman JS, Van der Molen HF, Frings-Dresen MH, Sluiter JK. Preventive actions taken by workers after workers' health surveillance: a controlled trial. J Occup Environ Med. 2013;55(12):1401-8. | Included |  |
| 7 | Boschman JS, van der Molen HF, Sluiter JK, Frings-Dresen MH. Improving occupational health care for construction workers: a process evaluation. BMC Public Health. 2013;13:218. | Included |  |
| 8 | Choi KE, Lindert L, Schlomann L, Samel C, Hellmich M, Pfaff H. A cross-provider health care management program for musculoskeletal disorders: Results of a randomized controlled trial in 22 german companies. International Journal of Environmental Research and Public Health. 2021;18(22). | Excluded | No aspect of health surveillance feedback |
| 9 | Codling A, Executive S. Current practices in noise health surveillance An exploratory study on the delivery of noise health surveillance programmes in Britain. Occupational Health at Work 2. 2017;14(2):31-5. | Included |  |
| 10 | Cohen DA, Aylward M, Rollnick S. Inside the fitness for work consultation: a qualitative study. Occupational Medicine (Oxford). 2009;59(5):347-52. | Excluded | Not relevant objective |
| 11 | Cults S, Citera M. Individual feedback: does it enhance effects of group feedback? J Organizational Behavior Management. 1989;10:77-92. | Excluded | Not health surveillance in work life |
| 12 | Di Battista EM, Bracken RM, Stephens JW, Rice S, Williams SP, Thomas M, et al. Cardiovascular risk assessments at occupational health services: employee experiences. Occupational Medicine (Oxford). 2019;69(2):106-12. | Included |  |
| 13 | Dille JH. Worksite influenza immunization. Successful program. AAOHN Journal. 1999;47(7):292-300. | Excluded | Not relevant objective |
| 14 | Eaton JL, Mohr DC, Mohammad A, Kirkhorn S, Gerstel-Santucci C, McPhaul K, et al. Implementation of a Novel Occupational and Environmental Medicine Specialty Teleconsultation Service: The VHA Experience. Journal of Occupational and Environmental Medicine. 2015;57(2):173-7. | Excluded | Not relevant objective |
| 15 | Edwards A, Hood K, Matthews E, Russell D, Russell I, Barker J, et al. The effectiveness of one-to-one risk communication interventions in health care: a systematic review. Med Decis Making. 2000;20(3):290-7. | Excluded | Not relevant objective |
| 16 | Eklöf M, Burström L, Hagberg M, Holmberg K, Jonsson P, Lundström R, et al. Workplace intervention for improved risk perception and preventive activity among workers using hand-held vibrating machines: a pilot study. Folkhälsa och klinisk medicin i Umeå rapporterar 2023;(4):1-21. [http://umu.diva-portal.org](https://protect.checkpoint.com/v2/r02/___http:/umu.diva-portal.org___.YzJlOnJlZ2lvbnNrYW5lOmM6bzoyOTg5N2ZkYTIwMWNmNzhkYjMyMjQ4YjgzN2Q4M2I0Nzo3OjkyMDE6NjU4YWVlODk5MTdmNzI0Zjg2YTllZjYxODI0ZmEzODk5ZmYwYjkwMWQzNmJkNWFjYjJhMmNlZTEzZGU4ZjE0YjpoOlQ6Tg)/ | Excluded | Not relevant objective |
| 17 | Eklöf M, Hagberg M. Are simple feedback interventions involving workplace data associated with better working environment and health? A cluster randomized controlled study among Swedish VDU workers. Applied Ergonomics. 2006;37(2):201-10. | Included |  |
| 18 | Eklöf M, Hagberg M, Toomingas A, Tornqvist EW. Feedback of workplace data to individual workers, workgroups or supervisors as a way to stimulate working environment activity: a cluster randomized controlled study. International Archives of Occupational & Environmental Health. 2004;77(7):505-14. | Included |  |
| 19 | Eliasson K, Dahlgren G, Hellman T, Lewis C, Palm P, Svartengren M, et al. Company Representatives' Experiences of Occupational Health Surveillance for Workers Exposed to Hand-Intensive Work: A Qualitative Study. Int J Environ Res Public Health. 2021;18(4). | Included |  |
| 20 | Eliasson K, Fjellman-Wiklund A, Dahlgren G, Hellman T, Svartengren M, Nyman T, et al. Ergonomists' experiences of executing occupational health surveillance for workers exposed to hand-intensive work: a qualitative exploration. Bmc Health Services Research. 2022;22(1):1223. | Included |  |
| 21 | Elo AL, Leppanen A, Sillanpaa P. Applicability of survey feedback for an occupational health method in stress management. Occupational Medicine (Oxford). 1998;48(3):181-8. | Excluded | Not relevant objective |
| 22 | Fishwick D, Sen D, Barker P, Codling A, Fox D, Naylor S. Health surveillance for occupational asthma in the UK. Occupational Medicine. 2016;66(5):365-70. | Included |  |
| 23 | Franco G, Cella MT, Tuccillo E, Ferrari F, Minisci E, Fusetti L. From risk-based health surveillance to health promotion: an evidence-based experience in a health care setting. Int J Occup Med Environ Health. 2002;15(2):117-20. | Included |  |
| 24 | Gartner FR, Ketelaar SM, Smeets O, Bolier L, Fischer E, van Dijk FJ, et al. The Mental Vitality @ Work study: design of a randomized controlled trial on the effect of a workers' health surveillance mental module for nurses and allied health professionals. Bmc Public Health. 2011;11:290. | Excluded | No aspect of health surveillance feedback |
| 25 | Goetzel RZ, Henke RM, Head MA, Benevent R, Calitz C. Workplace Programs, Policies, And Environmental Supports To Prevent Cardiovascular Disease. Health Affairs. 2017;36(2):229-36. | Excluded | Wrong outcomes |
| 26 | Groeneveld IF, Proper KI, van der Beek AJ, van Duivenbooden C, van Mechelen W. Design of a RCT evaluating the (cost-) effectiveness of a lifestyle intervention for male construction workers at risk for cardiovascular disease: the health under construction study. BMC Public Health. 2008;8:1. | Included |  |
| 27 | Grooten WJ, Muller M, Forsman M, Kjellberg K, Toomingas A, Bjorn Olov A, et al. Health risk appraisals in Swedish occupational health services. Work. 2016;55(4):849-59. | Included |  |
| 28 | Hanlon P, Carey L, Tannahill C, Kelly M, Gilmour H, Tannahill A, et al. Behaviour change following a workplace health check: how much change occurs and who changes? Health Promotion International. 1998;13(2):131-9. | Included |  |
| 29 | Heirich M, Sieck CJ. Worksite cardiovascular wellness programs as a route to substance abuse prevention. Journal of Occupational & Environmental Medicine. 2000;42(1):47-56. | Excluded | Not relevant objective |
| 30 | Hendriksen IJM, Snoijer M, De Kok BPH, Van Vilsteren J, Hofstetter H. Effectiveness of a Multilevel Workplace Health Promotion Program on Vitality, Health, and Work-Related Outcomes. Journal of Occupational and Environmental Medicine. 2016;58(6):575-83. | Excluded | Not relevant objective |
| 31 | Hoge A, Ehmann AT, Rieger MA, Siegel A. Caring for Workers' Health: Do German Employers Follow a Comprehensive Approach Similar to the Total Worker Health Concept? Results of a Survey in an Economically Powerful Region in Germany. International Journal of Environmental Research & Public Health [Electronic Resource]. 2019;16(5):28. | Excluded | Not relevant objective |
| 32 | Hogg B, Moreno-Alcazar A, Toth MD, Serbanescu I, Aust B, Leduc C, et al. Supporting employees with mental illness and reducing mental illness-related stigma in the workplace: an expert survey. European Archives of Psychiatry and Clinical Neuroscience. 2022. | Excluded | Wrong outcomes |
| 33 | Holmgren K, Hensing G, Bultmann U, Hadzibajramovic E, Larsson MEH. Does early identification of work-related stress, combined with feedback at GP-consultation, prevent sick leave in the following 12 months? a randomized controlled trial in primary health care. BMC Public Health. 2019;19(1):1110. | Excluded | Not health surveillance in work life |
| 34 | Hubbell Z, Howard S, Golden A, Stange B, Cragle D, Dally M, et al. Factors linked to participant attrition in a longitudinal occupational health surveillance program. Am J Ind Med. 2022;65(6):431-46. | Included |  |
| 35 | Jetha A, Le Pouesard M, Mustard C, Backman C, Gignac MAM. Getting the Message Right: Evidence-Based Insights to Improve Organizational Return-to-Work Communication Practices. Journal of Occupational Rehabilitation. 2021;31(3):652-63. | Excluded | Not health surveillance, only risk factors |
| 36 | Jetzer T, Haydon P, Reynolds D. Effective intervention with ergonomics, antivibration gloves, and medical surveillance to minimize hand-arm vibration hazards in the workplace. J Occup Environ Med. 2003;45(12):1312-7. | Excluded | No aspect of health surveillance feedback |
| 37 | Kaatz M, Ladermann R, Stadeler M, Fluhr JW, Elsner P, Bauer A. Recruitment strategies for a hand dermatitis prevention programme in the food industry. Contact Dermatitis. 2008;59(3):165-70. | Excluded | Not health surveillance in work life |
| 38 | Ketelaar SM, Gartner FR, Bolier L, Smeets O, Nieuwenhuijsen K, Sluiter JK. Mental Vitality @ Work--a workers' health surveillance mental module for nurses and allied health care professionals: process evaluation of a randomized controlled trial. Journal of Occupational & Environmental Medicine. 2013;55(5):563-71. | Included |  |
| 39 | Ketelaar SM, Nieuwenhuijsen K, Bolier L, Smeets O, Sluiter JK. Improving work functioning and mental health of health care employees using an e-mental health approach to workers' health surveillance: Pretest-posttest study. Safety and Health at Work. 2014;5(4):216-21. | Included |  |
| 40 | Kluger AN, Van Dijk D. Feedback, the various tasks of the doctor, and the feedforward alternative. Med Educ. 2010;44(12):1166-74. | Excluded | Not health surveillance feedback results |
| 41 | Linton SJ, Boersma K, Traczyk M, Shaw W, Nicholas M. Early Workplace Communication and Problem Solving to Prevent Back Disability: Results of a Randomized Controlled Trial Among High-Risk Workers and Their Supervisors. Journal of Occupational Rehabilitation. 2016;26(2):150-9. | Excluded | Not relevant objective |
| 42 | Los FS, de Boer A, van der Molen HF, Hulshof CTJ. The Implementation of Workers' Health Surveillance by Occupational Physicians: A Survey Study. J Occup Environ Med. 2019;61(12):e497-e502. | Excluded | Not health surveillance feedback results |
| 43 | Los, F. S.; van der Molen, H. F.; Hulshof, C. T. J.; de Boer, A.G.E.M. Supporting Occupational Physicians in the Implementation of Workers' Health Surveillance: Development of an Intervention Using the Behavior Change Wheel Framework. International Journal of Environmental Research & Public Health [Electronic Resource] 02 17 2021;18(4):17. | Excluded | Not relevant objective |
| 44 | Magnavita N. Workplace Health Promotion Embedded in Medical Surveillance: The Italian Way to Total Worker Health Program. International Journal of Environmental Research & Public Health [Electronic Resource]. 2023;20(4):18. | Included |  |
| 45 | Maizlish N, Rudolph L, Dervin K, Sankaranarayan M. Surveillance and prevention of work-related carpal tunnel syndrome: an application of the Sentinel Events Notification System for Occupational Risks. Am J Ind Med. 1995;27(5):715-29 | Included |  |
| 46 | Maron DJ, Forbes BL, Groves JR, Dietrich MS, Sells P, DiGenio AG. Health-risk appraisal with or without disease management for worksite cardiovascular risk reduction. Journal of Cardiovascular Nursing. 2008;23(6):513-8. | Excluded | Not relevant objective |
| 47 | Marsh GM, Leviton LC, Talbott EO, Callahan C, Pavlock D, Hemstreet G, et al. Drake Chemical Workers' Health Registry Study: I. Notification and medical surveillance of a group of workers at high risk of developing bladder cancer. Am J Ind Med. 1991;19(3):291-301. | Included |  |
| 48 | Masci F, Spatari G, Giorgianni CM, Pernigotti E, Antonangeli LM, Bordoni V, et al. Hand-Wrist Disorders in Chainsaw Operators: A Follow-Up Study in a Group of Italian Loggers. Int J Environ Res Public Health. 2021;18(14). | Excluded | Not health surveillance feedback results |
| 49 | Mazurek JM, Storey E. Physician-patient communication regarding asthma and work. American Journal of Preventive Medicine. 2012;43(1):72-5. | Excluded | Wrong outcomes |
| 50 | Mazurek JM, White GE, Moorman JE, Storey E. Patient-physician communication about work-related asthma: what we do and do not know. Annals of Allergy, Asthma, & Immunology. 2015;114(2):97-102. | Excluded | Not relevant objective |
| 51 | McMahan S, Meyer J. Communication of risk information to workers and managers: Do industrial hygienists differ in their communication techniques? American Industrial Hygiene Association Journal. 1996;57(2):186-90. | Excluded | Not health surveillance in work life |
| 52 | Menckel E, Hagberg M, Engkvist I, Wigaeus Hjelm EW. The prevention of back injuries in Swedish health care - a comparison between two models for action-oriented feedback. Applied Ergonomics. 1997;28(1):1-7. | Included |  |
| 53 | Naumanen P. The health promotion of aging workers from the perspective of occupational health professionals. Public Health Nursing. 2006;23(1):37-45. | Excluded | Not relevant objective |
| 54 | Nicholson PJ, Mayho GV, Roomes D, Swann AB, Blackburn BS. Health surveillance of workers exposed to laboratory animal allergens. Occupational Medicine (Oxford). 2010;60(8):591-7. | Excluded | Wrong patient population |
| 55 | Nuraydin A, Bilek O, Kenziman AK, Korkusuz MA, Atagun AI, Cakar NO, et al. The Mersin Greenhouse Workers Study. Surveillance of Work-related Skin, Respiratory, and Musculoskeletal Diseases. Annals of Global Health. 2018;84(3):504-11. | Excluded | Not relevant objective |
| 56 | Perbellini L, Di Leo E, Goio I. The occupational physician and communication to workers. [Italian]. Giornale Italiano di Medicina del Lavoro Ed Ergonomia. 2010;32(4):412-4. | Excluded | Not in English/Scandinavian language |
| 57 | Plat MJ, Frings-Dresen MH, Sluiter JK. Feasibility and acceptability of workers' health surveillance for fire fighters. Saf Health Work. 2011;2(3):218-28. doi: DOI: 10.5491/SHAW.2011.2.3.218. PubMed PMID: 22953205 PubMed Central PMCID: PMCPMC3430907. | Included |  |
| 58 | Poole K, Mason HJ, Harris-Roberts J. Uptake and quality of health surveillance for noise and hand–arm vibration. Occupational Medicine. 2011;61(5):354-6. | Excluded | Not relevant objective |
| 59 | Pransky GS, Shaw WS, Franche RL, Clarke A. Disability prevention and communication among workers, physicians, employers, and insurers - Current models and opportunities for improvement. Disability and Rehabilitation. 2004;26(11):625-34. | Excluded | Not health surveillance in work life |
| 60 | Rantonen J, Vehtari A, Karppinen J, Luoto S, Viikari-Juntura E, Hupli M, et al. Face-to-face information combined with a booklet versus a booklet alone for treatment of mild low-back pain: A randomized controlled trial. Scandinavian Journal of Work, Environment and Health. 2014;40(2):156-66. | Excluded | Not health surveillance in work life |
| 61 | Robroek SJ, van de Vathorst S, Hilhorst MT, Burdorf A. Moral issues in workplace health promotion. International Archives of Occupational & Environmental Health. 2012;85(3):327-31. | Included |  |
| 62 | Ruitenburg MM, Plat MC, Frings-Dresen MH, Sluiter JK. Feasibility and acceptability of a workers' health surveillance program for hospital physicians. Int J Occup Med Environ Health. 2015;28(4):731-9. | Included |  |
| 63 | Saiki CL, Green RS, Gold EB, Schenker MB. Communication issues in a multicomponent study of semiconductor employees. American Journal of Industrial Medicine. 1995;28(6):883-911. | Excluded | Not health surveillance in work life |
| 64 | Sen S, Barlas G, Yakistiran S, Derin IG, Serifi BA, Ozlu A, et al. Prevention of Occupational Diseases in Turkey: Deriving Lessons From Journey of Surveillance. Sh@w. 2019;10(4):420-7. | Excluded | Not relevant objective |
| 65 | Seneviratne M, Shankar K, Cantrell P, Nand A. Respirable crystalline silica (RCS) exposure monitoring, health surveillance and hazard communication in preventing silicosis among stone workers. Occupational and Environmental Medicine. 2018;75(Supplement 2):A441. | Excluded | Not full text article |
| 66 | Seward JP. Medical surveillance of allergy in laboratory animal handlers. Ilar j. 2001;42(1):47-54. | Included |  |
| 67 | Soler RE, Leeks KD, Razi S, Hopkins DP, Griffith M, Aten A, et al. A systematic review of selected interventions for worksite health promotion. The assessment of health risks with feedback. American Journal of Preventive Medicine. 2010;38(2 Suppl):S237-62. | Included |  |
| 68 | Steel JS, Godderis L, Luyten J. Disclosure in Online vs. Face-to-Face Occupational Health Screenings: A Cross-Sectional Study in Belgian Hospital Employees. International Journal of Environmental Research & Public Health [Electronic Resource]. 2021;18(4):04. | Included |  |
| 69 | Stern AF, Madan I. Optimal communication from occupational physicians to GPs: a cross-sectional survey. British Journal of General Practice. 2012;62(605):e833-9. | Excluded | Not relevant objective |
| 70 | Street TD, Lacey SJ. Employee Perceptions of Workplace Health Promotion Programs: Comparison of a Tailored, Semi-Tailored, and Standardized Approach. International Journal of Environmental Research & Public Health [Electronic Resource]. 2018;15(5):28. | Excluded | Not health surveillance in work life |
| 71 | Talvi AI, Jarvisalo JO, Knuts LR. A health promotion programme for oil refinery employees: Changes of health promotion needs observed at three years. Occupational Medicine. 1999;49(2):93-101. | Excluded | Not relevant objective |
| 72 | Tarride JE, Harrington K, Balfour R, Simpson P, Foord L, Anderson L, et al. Evaluation of a workplace health program for British Columbia public service agency (Canada) - An example of partnership in employee health. Journal of Population Therapeutics and Clinical Pharmacology. 2011;18(2):e194-e5. | Excluded | Wrong outcomes |
| 73 | Udasin IG, Buckler G, Gochfeld M. Quality assurance audits of medical surveillance programs for hazardous waste workers. J Occup Med. 1991;33(11):1170-4. | Excluded | Not health surveillance feedback results |
| 74 | van Doorn D, Richardson N, Osborne A, Blake C. The impact of a workplace cardiovascular health screening programme 'Farmers Have Hearts' on health behaviour change among Irish farmers. Work. 2019;63(1):113-23. | Excluded | Not relevant objective |
| 75 | Vernon SW, Tilley BC, Myers R, Glanz K, Lu M, Hirst K, et al. The next step trial: Impact of a worksite colorectal cancer screening promotion program. American Journal of Epidemiology. 1998;147(11):S39-S. | Excluded | Not health surveillance feedback results |
| 76 | Vigna L, Barberi C, Conti D, Consonni D, Bordini L, Nava C, et al. Healthy lifestyle promotion among workers of a major hospital in northern Italy. European Journal of Preventive Cardiology. 2017;24(2 Supplement 1):46-7. | Excluded | Not relevant objective |
| 77 | van Holland BJ, Reneman MF, Soer R, Brouwer S, de Boer MR. Effectiveness and Cost-benefit Evaluation of a Comprehensive Workers' Health Surveillance Program for Sustainable Employability of Meat Processing Workers. Journal of Occupational Rehabilitation. 2018;28(1):107-20. | Included |  |
| 78 | Warner CH, Appenzeller GN, Grieger T, Belenkiy S, Breitbach J, Parker J, et al. Importance of Anonymity to Encourage Honest Reporting in Mental Health Screening After Combat Deployment. Archives of General Psychiatry. 2011;68(10):1065-71. | Excluded | Not relevant objective |
| 79 | Welch L, Roto P. Medical surveillance programs for construction workers. Occup Med. 1995;10(2):421-33. | Excluded | Not relevant objective |
| 80 | Verweij LM, Proper KI, Weel AN, Hulshof CT, van Mechelen W. Long-term effects of an occupational health guideline on employees' body weight-related outcomes, cardiovascular disease risk factors, and quality of life: results from a randomized controlled trial. Scandinavian Journal of Work, Environment & Health. 2013;39(3):284-94. | Included |  |
| 81 | Williams W, Purdy SC, Murray N, Dillon H, Lepage E, Challinor K, et al. Does the presentation of audiometric test data have a positive effect on the perceptions of workplace noise and noise exposure avoidance? Noise and Health. 2004;6(24):75-84. | Included |  |
| 82 | Wright-Hughes A, Willis TA, Wilson S, Weller A, Lorencatto F, Althaf M, et al. A randomised fractional factorial screening experiment to predict effective features of audit and feedback. Implement Sci. 2022;17(1):34. | Excluded | Not health surveillance in work life |
| 83 | Zarate P, Cuellar D, Velazquez L, Cura L. Occupational health nurses working as worksite health promotion agents. Occupational and Environmental Medicine. 2018;75(Supplement 2):A165. | Excluded | Not full text article |
| 84 | Zohar D, Cohen A, Azar N. Promoting increased use of ear protectors in noise through information feedback. Hum Factors. 1980;22(1):69-79. | Included |  |
