# Supplementary figures and images for: Communication aspects of feedback from workers’ health surveillance due to hand-arm vibration exposure − a scoping review
Source: J Occup Med Toxicol. 2025 May 21;20:16. doi: 10.1186/s12995-025-00463-8 (PMC12093587; doi:10.1186/s12995-025-00463-8)

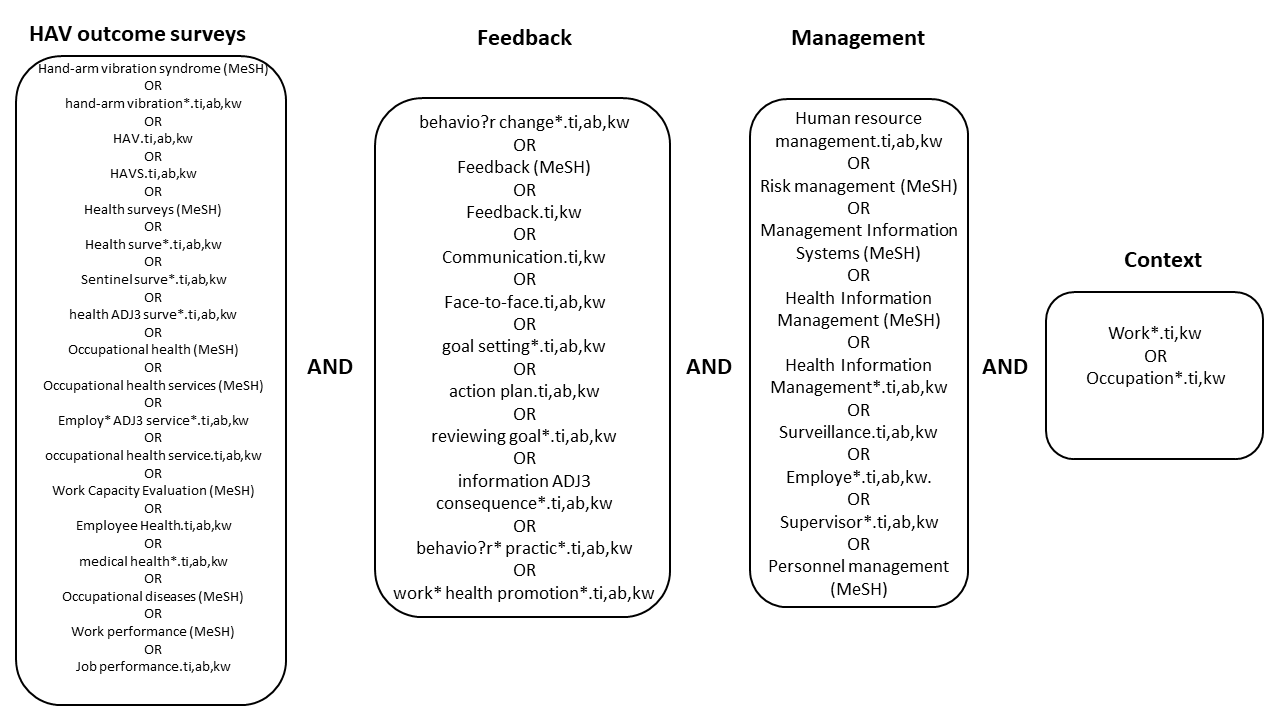

Supplement: Supplementary file 2 — Supplementary Material 2: Fig. 1. The final search strategy consisted of four blocks at database search in Ovid MEDLINE®, Embase® Classic and Embase®, conditionally combined using Boolean operators. [file 12995_2025_463_MOESM2_ESM.tif]

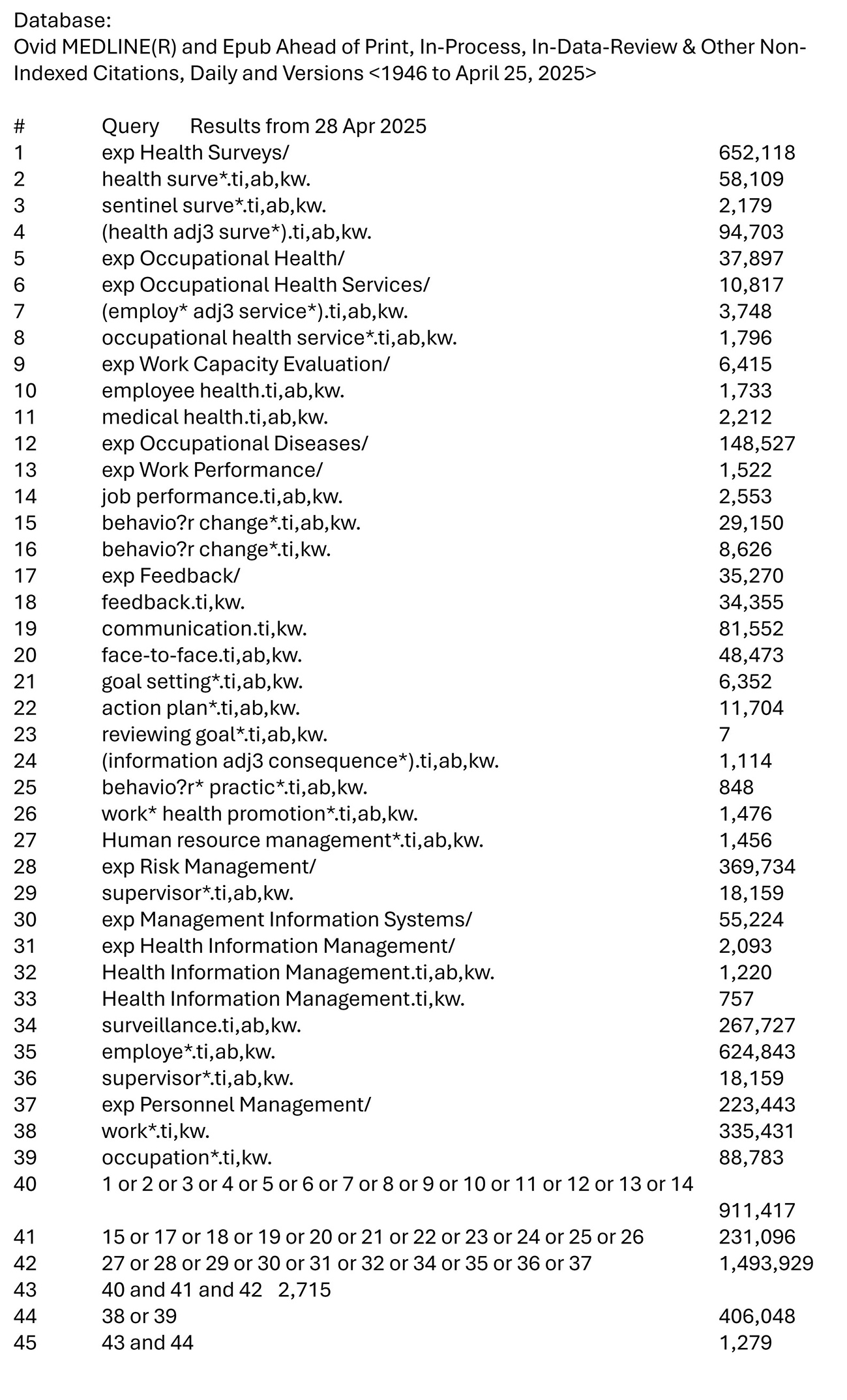

Supplement: Supplementary file 3 — Supplementary Material 3: Fig. 2. Example search string Medline. [file 12995_2025_463_MOESM3_ESM.jpg]
